# Trends in Primary- and Metastasis-Directed Radiotherapy Uptake and Survival in Metastatic Prostate Cancer

**DOI:** 10.3390/jcm15083010

**Published:** 2026-04-15

**Authors:** James O. Suggitt, Tej A. Patel, Miranda B. Lam, Irini Yacoub, Puneeth Iyengar, Sean M. McBride, Himanshu Nagar, Paul L. Nguyen, Daniel Gorovets, Jonathan E. Leeman, Edward Christopher Dee

**Affiliations:** 1 Department of Radiation Oncology, Grossman School of Medicine, New York University, New York, NY 10016, USA; james.suggitt@nyulangone.org; 2 Nuffield Department of Population Health, University of Oxford, Oxford OX3 7LF, UK; 3 Department of Radiation Oncology, Dana Farber Cancer Institute, Brigham and Women’s Hospital, Boston, MA 02215, USAjonathane_leeman@dfci.harvard.edu (J.E.L.); 4 Department of Health Policy and Management, Harvard T.H. Chan School of Public Health, Boston, MA 02115, USA; 5 New York Proton Center, New York, NY 10035, USA; 6 Department of Radiation Oncology, Memorial Sloan Kettering Cancer Center, New York, NY 10065, USA

**Keywords:** metastatic prostate cancer, genitourinary cancer, radiotherapy, primary-directed radiotherapy, metastasis-directed radiotherapy, radiotherapy uptake trends

## Abstract

**Background/Objectives**: This study investigates trends in radiotherapy utilization in men with metastatic prostate cancer, particularly in response to pivotal trials like STAMPEDE and STOMP, which demonstrated survival benefits for primary- and metastasis-directed radiotherapy. **Methods**: Using data from the National Cancer Database, the study analyses changes in treatment patterns post-2018. **Results**: Prior to 2018, 13.3% and 10.9% of patients with metastatic prostate cancer received primary- and metastasis-directed radiotherapy, respectively; these proportions increased modestly to 14.0% and 12.1% following 2018 (χ^2^ *p* < 0.0001 for both). Significant differences in comorbidity prevalence were observed both temporally (CDCI mean 0.35 prior to 2018 vs. 0.46 post-2018, *p* < 0.0001), and between those treated with primary- and metastasis-directed radiotherapy (CDCI mean 0.34 in primary-directed vs. 0.45 in metastasis-directed, *p* < 0.0001). Primary-directed radiotherapy was associated with improved overall survival across all years (HR 0.87, *p* < 0.0001), but it was associated with lower survival when restricted to post-2018 diagnoses (HR 1.24, *p* < 0.0001). Metastasis-directed radiotherapy was not significantly associated with overall survival benefit regardless of era (M1a: HR 1.58, *p* = 0.27; M1b: HR 0.97, *p* = 0.29), though non-receipt after 2018 was associated with markedly increased mortality (HR 18.54, *p* < 0.0001)**. Conclusions**: Pivotal clinical trials temporally align with shifts in radiotherapy practices among M1 disease subgroups. The potential survival benefit of radiotherapy to the primary site and to metastases among patients with metastatic disease merits further investigation in the real-world setting. Further work may highlight differences in patient populations or overall efficacy of radiotherapy in these populations.

## 1. Introduction

Prostate cancer is the most common cancer among men in the United States, with mortality concentrated in patients with metastatic disease [1]. Radiotherapy remains a cornerstone of treatment, and recent large-scale trials—including STAMPEDE [2], STOMP [3], PEACE01 [4], and others—have shown survival benefits for primary- and metastasis-directed approaches. STAMPEDE included 2061 patients with newly diagnosed prostate cancer, with metastatic disease and intended long-term androgen deprivation therapy, who had not received a radical prostatectomy. Randomizing patients to standard of care with or without radiotherapy to the prostate, the investigators selected overall survival as a primary endpoint, with failure-free survival used for interim analyses. Radiotherapy was found to improve failure-free survival, but not overall survival. STOMP provided surveillance or metastasis-directed therapy to 62 patients with asymptomatic biochemical recurrence. With a primary endpoint of androgen deprivation therapy-independent survival, metastasis-directed radiotherapy demonstrating increased median time to androgen deprivation therapy. Initially published in 2018 (STAMPEDE and STOMP), these studies may have influenced later clinical practice.

This study evaluates whether treatment patterns in metastatic prostate cancer have shifted in response to these findings and examines whether these changes are associated with improved overall survival.

## 2. Materials and Methods

Patients diagnosed with prostate cancer between 2004 and 2021 were identified from the National Cancer Database (NCDB, retrieved 2025, American College of Surgeons, Chicago, IL, USA), which accounts for ~70% of US cancer diagnoses [5]. Stage at presentation was defined using contemporary AJCC criteria. Included patients had complete data on radiotherapy receipt, disease stage, race, and treatment region. Radiotherapy receipt was defined as a recorded radiation volume in any phase; the analytic stage group combined pathological and clinical TNM stage, preferencing pathological diagnosis; race was aggregated (White, Black, Native American, Asian and Pacific Islander); and the encoded site in radiotherapy volume (specific organ, lymph node region, or regional) was used to determine disease site, respectively.

The NCDB contains data regarding radiotherapy targets. Radiotherapy to the primary tumor was defined as radiation to any portion of the prostate or pelvic lymph nodes. Radiotherapy to other lymph node regions alone indicated M1a disease, while radiotherapy directed to any portion of the skeleton indicated M1b disease, with M1c disease defined as radiotherapy to any field outside of previously specified fields. Evaluation assigned each patient the most advanced classification among the reported radiotherapy volumes. Patients were stratified by metastatic burden, with patients presenting with M1c disease excluded.

Primary- and metastasis-directed uptake was evaluated using logistic regression models, with patients not receiving radiotherapy as the reference group. Covariates included age, year of diagnosis, chemotherapy and hormone therapy receipt, race, insurance status, Charlson–Deyo Comorbidity Index (CDCI), facility type, zip-code based income, education and distance to treatment facility.

Overall survival was analyzed using a Cox proportional hazards model, with patients not receiving radiotherapy as the reference group. Covariates included age, year of diagnosis, chemotherapy and hormone therapy receipt, facility type, race, insurance status, CDCI, and zip code-based income, education, and distance to treatment facility. Zip code-level deidentification limited individual-level socioeconomic analysis.

All analyses were conducted in R (v4.4.3; 2025, R Foundation, Vienna, Austria). This study was exempt from institutional review board oversight due to the use of deidentified, publicly available data.

## 3. Results

### 3.1. Baseline Characteristics

Of the 2,161,253 patients in the NCDB, 67,151 met the inclusion criteria. Within this group, the majority were White (77.2%). M1b disease was most common (92.2%), followed by M1a (7.8%). In total, 12.2% of patients received chemotherapy, while 82.6% received hormone therapy (Table 1). The relative comorbidity burden was low (73.7% with CDCI = 0). Both groups receiving primary- and metastasis-directed radiotherapy were enriched for M1a and M1b disease and hormone therapy administration (χ^2^ *p* < 0.0001 for all).

In the entire cohort, comorbidity burden differed by year of diagnosis, with patients diagnosed prior to 2018 demonstrating a mean CDCI 0.11 points lower than those diagnosed after 2018 (0.35 vs. 0.46). Comparing radiotherapy administration, those receiving metastasis-directed radiotherapy had an increased mean CDCI both prior to (0.37 vs. 0.23) and after 2018 (0.45 vs. 0.34), when compared to primary-directed radiotherapy (Welch’s two sample *t*-test, *p* < 0.0001 for all).

### 3.2. Uptake Trends

**Figure 1:**
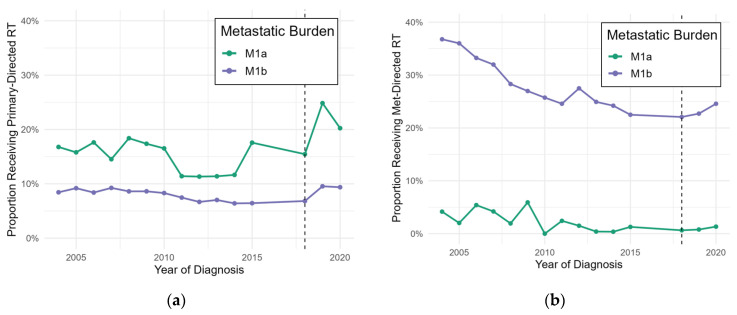
Trends in radiotherapy directed at the primary tumor (**a**) or metastases (**b**) from 2004 to 2021, stratified by disease subtype. The year of release of relevant data (2018) is indicated by the dashed line.

The proportion of patients receiving radiation to the primary tumor remained relatively low prior to 2018 (Figure 1a). After 2018, patients were significantly more likely to receive primary-directed radiotherapy (OR 1.91, *p* < 0.0001). However, patients diagnosed with M1b disease were less likely to receive primary directed radiotherapy both overall (OR 0.49, *p* < 0.0001) and after 2018 (OR 0.70, *p* = 0.0001). While hormone therapy was associated with an increased likelihood of primary-directed radiotherapy (OR 2.13, *p* < 0.0001), chemotherapy was not.

Metastasis-directed radiotherapy remained relatively infrequent in patients with M1a disease and exhibited an apparent decline in M1b disease prior to 2018 (Figure 1b). Prior to 2018, among patients with metastatic disease, 13.3% received primary-directed radiotherapy and 10.9% received metastasis-directed radiotherapy. Following 2018, 14.0% received primary-directed radiotherapy, and 12.1% received metastasis-directed radiotherapy (χ^2^ *p* < 0.0001 for both). There is no significant difference in the likelihood of radiotherapy receipt for either subgroup after 2018, but hormone therapy is again associated with an increased likelihood of radiotherapy receipt (OR 1.62, *p* < 0.0001).

When compared with patients not receiving radiation, receiving primary-directed radiotherapy was significantly associated with improved survival (Table 2). However, considering patients diagnosed after 2018, receiving primary-directed radiotherapy was associated with worsened survival (Table 2). When stratified by metastatic burden, more advanced disease was associated with increased mortality (Table 2).

In comparison to no radiotherapy, across all years of diagnosis, there was no significant difference in hazard of mortality (M1a: HR 1.58, *p* = 0.27; M1b: HR 0.97 *p* = 0.29) with receipt of metastasis-directed radiotherapy. Among patients treated with metastasis-directed radiotherapy, treatment post-2018 was not associated with improved survival (M1a: HR 0.50, *p* = 0.33; M1b: HR 1.07, *p* = 0.11). Notably, among patients diagnosed after 2018 who were not treated with metastasis-directed radiotherapy, an increased likelihood of mortality was observed (HR 18.8, *p* < 0.0001).

## 4. Discussion

Patterns of radiotherapy utilization in metastatic prostate cancer have shifted since 2018, with increasing uptake of both primary- and metastasis-directed approaches. These changes align temporally with the publication of STAMPEDE and STOMP that demonstrated survival benefits for radiotherapy in select metastatic populations [2,3]. Our findings suggest that these studies may have influenced clinical practice in corresponding M1 subgroups.

We observed a marked increase in the use of primary-directed radiotherapy following 2018, with a simultaneous reduction in utilization in M1b disease. While uptake remained relatively low before 2018, these findings may indicate growing confidence in the role of local therapy in the metastatic setting. Across the cohort, patients with M1b disease were substantially more likely to receive metastasis-directed radiotherapy, which may reflect both the recent demonstrated survival advantages of metastasis-directed radiotherapy and the symptomatic relief that bone-targeted radiotherapy can provide [6]. As hormone therapy receipt was associated with radiotherapy utilization, this suggests that uptake may be concentrated among patients deemed fit enough to receive systemic treatment.

Despite changes in the patterns of care delivery, survival outcomes appear heterogenous. Receipt of primary-directed radiotherapy was associated with improved survival in the entire cohort, yet when restricted to patients treated after 2018, the same treatment displays a paradoxically worsened survival. Notably, the mortality risk is further increased in patients not receiving any primary-directed radiotherapy after 2018. In contrast, metastasis-directed radiotherapy demonstrated no significant difference in mortality compared to no radiotherapy, whether considering the entire cohort or those treated after 2018. Yet, there remains a similar substantial elevation in mortality risk not receiving metastasis-directed radiotherapy. These results may reflect the role of radiotherapy in palliative care or a preference for radiotherapy in patients unable to receive systemic treatment. Furthermore, it may be that patients with increased comorbidities or greater metastatic burden not fully captured in the NCDB were considered for primary-directed RT following STAMPEDE.

Additionally, the COVID-19 pandemic may have disproportionately impacted both initial cancer care and long-term follow-up for patients diagnosed after 2018, which, coupled with the NCDB’s documented struggles with long-term survival follow-up, may contribute to the observed trends [7], as patients lost to follow-up do not provide survival data.

Further, a shift in the patient population treated with radiotherapy may obscure survival benefits. Given that STAMPEDE [2] was conducted on newly diagnosed prostate cancer and STOMP [3] focused on asymptomatic recurrence, this may not be indicative of effects in the population of patients recommended radiotherapy. The increased risk in mortality associated with non-receipt of radiotherapy post-2018 may suggest that patients with more advanced disease or greater overall metastatic burden are being offered radiotherapy, thus shifting the demographics of the patient population. While covariates for systemic therapy and CDCI were utilized to minimize this impact, the lack of more granular patient-level data precludes further stratification. As mean comorbidity burden differed significantly both temporally and between metastasis- and primary-directed radiotherapy, analyzing survival data may be complicated by this baseline disparity in the patient population. As such, patients with an increased burden of comorbid conditions may have been offered radiotherapy at higher rates post-2018 and may have been more likely to be treated with metastasis-directed radiotherapy. While controlling for socioeconomic and baseline functional status disparities, there may be a remaining patient-level difference in demographics which the NCDB is unable to identify.

## 5. Conclusions

Ultimately, following improvements in radiotherapy regimens, clinical practice in metastatic prostate cancer appears to have shifted, with a marked increase in primary-directed radiotherapy after the 2018 publication of STAMPEDE [2] and STOMP [3]. The survival benefit of these regimens remains uncertain, given differences in patient populations; however, non-receipt of radiotherapy after the introduction of these strategies is associated with notably worsened survival.

Due to the deidentified nature of the NCDB, it is not possible to effectively determine whether this change in patient volume has impacted the composition of the patient population beyond differences in comorbidity burden. Further work is required to determine whether shifting patterns in outcomes are attributable to differences in the patient populations receiving radiotherapy or in the efficacy of such treatments.

## Figures and Tables

**Table 1:** Patient sociodemographic and treatment characteristics of patients with metastatic prostate cancer diagnosed between 2004 and 2021.

Patient Characteristic	No. (%)
Metastatic Burden	
M1a	5197 (7.8)
M1b	61,954 (92.2)
Race	
White	51,858 (77.2)
Black	12,069 (18.0)
Native American	222 (0.3)
Asian	1128 (1.7)
Pacific Islander	152 (0.2)
Other	1722 (2.6)
Chemotherapy Receipt	
None	56,216 (83.7)
Multiple Doses	8152 (12.2)
Not Specified	2783 (4.1)
Hormone Therapy Receipt	
None	9789 (14.6)
Multiple Doses	55,455 (82.6)
Not Specified	1907 (2.8)
Charlson–Deyo Comorbidity Index	
0	49,490 (73.7)
1	10,352 (15.4)
2	4290 (6.4)
3	3019 (4.5)

**Table 2:** Overall survival by primary-directed radiotherapy receipt, stratified by metastatic burden.

Variable	Adjusted HR (95% CI)	*p*-Value
RT Receipt *		
None (2004–2017)	Ref	
None (after 2018)	16.54 (15.69–17.42)	<0.0001
Multiple Fractions (2004–2017)	0.87 (0.80–0.93)	<0.0001
Multiple Fractions (after 2018)	1.24 (1.12–1.36)	<0.0001
Metastatic Burden		
M1a	Ref	
M1b	1.09 (1.04–1.15)	0.001
Charlson–Deyo Comorbidity Index		
0	Ref	
1	1.04 (0.99–1.09)	0.13
2	1.12 (1.03–1.21)	0.006
≥3	1.15 (1.05–1.26)	0.002
Chemotherapy Receipt		
None	Ref	
Multiple Doses	0.87 (0.83–0.91)	<0.0001
Hormone Therapy Receipt		
None	Ref	
Multiple Doses	0.90 (0.85–0.95)	0.001
Age		
≤49	Ref	
49–64	1.08 (0.95–1.22)	0.27
≥65	1.20 (1.05–1.37)	0.01
Race		
White	Ref	
Black	1.05 (1.01–1.10)	0.04
Native American	0.83 (0.60–1.15)	0.26
Asian	1.01 (0.90–1.13)	0.90
Pacific Islander	1.26 (0.89–1.78)	0.19
Insurance Status		
Not Insured	Ref	
Private Insurance/Managed Care	0.80 (0.74–0.89)	<0.0001
Medicaid	0.99 (0.89–1.09)	0.84
Medicare	0.82 (0.75–0.89)	<0.0001
Other Government	0.96 (0.82–1.12)	0.58
Unknown	0.92 (0.78–1.08)	0.30
Income Quartile (median)		
<$46,277	Ref	
$46,277–$57,856	1.03 (0.97–1.08)	0.39
$57,857–$74,062	1.05 (0.99–1.11)	0.11
>$74,063	1.06 (1.01–1.13)	0.04
No High-School Degree		
>15.3%	Ref	
9.1–15.2%	0.92 (0.88–0.97)	0.001
5.0–9.0%	0.87 (0.83–0.92)	<0.0001
<5.0%	0.88 (0.83–0.93)	<0.0001

* Radiotherapy receipt was analyzed utilizing an interaction variable between radiotherapy receipt and year of diagnosis, comparing patients diagnosed after 2018 to those diagnosed before 2018.

## Data Availability

Restrictions apply to the availability of these data. Data were obtained from the National Cancer Database and are available with the permission of the American College of Surgeons.
